# Anomalous Pancreaticobiliary Duct Junction in an Unusual Case of Synchronous Gallbladder and Bile Duct Malignancy

**DOI:** 10.7759/cureus.13331

**Published:** 2021-02-13

**Authors:** Souradeep Dutta, Ankit Jain, Abhinaya Reddy, Vishnu Prasad Nelamangala Ramakrishnaiah

**Affiliations:** 1 Surgery, Jawaharlal Institute of Postgraduate Medical Education and Research, Puducherry, IND; 2 Surgery, Jawaharlal Institute of Postgraduate Medical Education and Research, Pondicherry, IND

**Keywords:** anomalous pancreaticobiliary duct junction, pancreaticobiliary maljunction, cholangiocarcinoma, gallbladder carcinoma

## Abstract

Synchronous malignancies involving the gallbladder and the bile duct are exceedingly rare. Moreover, their association with anomalous pancreaticobiliary duct junction (APBDJ) has been reported mostly from the Far Eastern countries. Over time, many studies have suggested the definite risk of malignancy attributable to the ‘carcinogenic anatomical configuration’ of the long common biliopancreatic channel, allowing reflux of pancreatic juices in the biliary tract. In this report, we present a case of an elderly man from South India who was initially diagnosed with synchronous gall bladder with bile duct malignancy; the patient turned out to have an APBDJ on further evaluation.

## Introduction

Synchronous malignancies involving the gallbladder and the bile duct are very rare [1], and mostly reported from the Far Eastern countries [2,3]. Various reasons for such synchronous occurrence have been postulated, such as an inherent multifocality of the primary tumor, the concept of field cancerization, metastasis, and anomalous pancreaticobiliary duct junction (APBDJ) [4]. Most cases of APBDJ have been reported from Japan. However, most case series reporting simultaneous gallbladder and bile duct cancer in the Indian population have stated other possible causes and not APBDJ as a causative factor [4,5]. Herein, we present the case of an elderly man with obstructive jaundice and was found to have a synchronous gall bladder and bile duct malignancy with APBDJ.

## Case presentation

**Figure 1:**
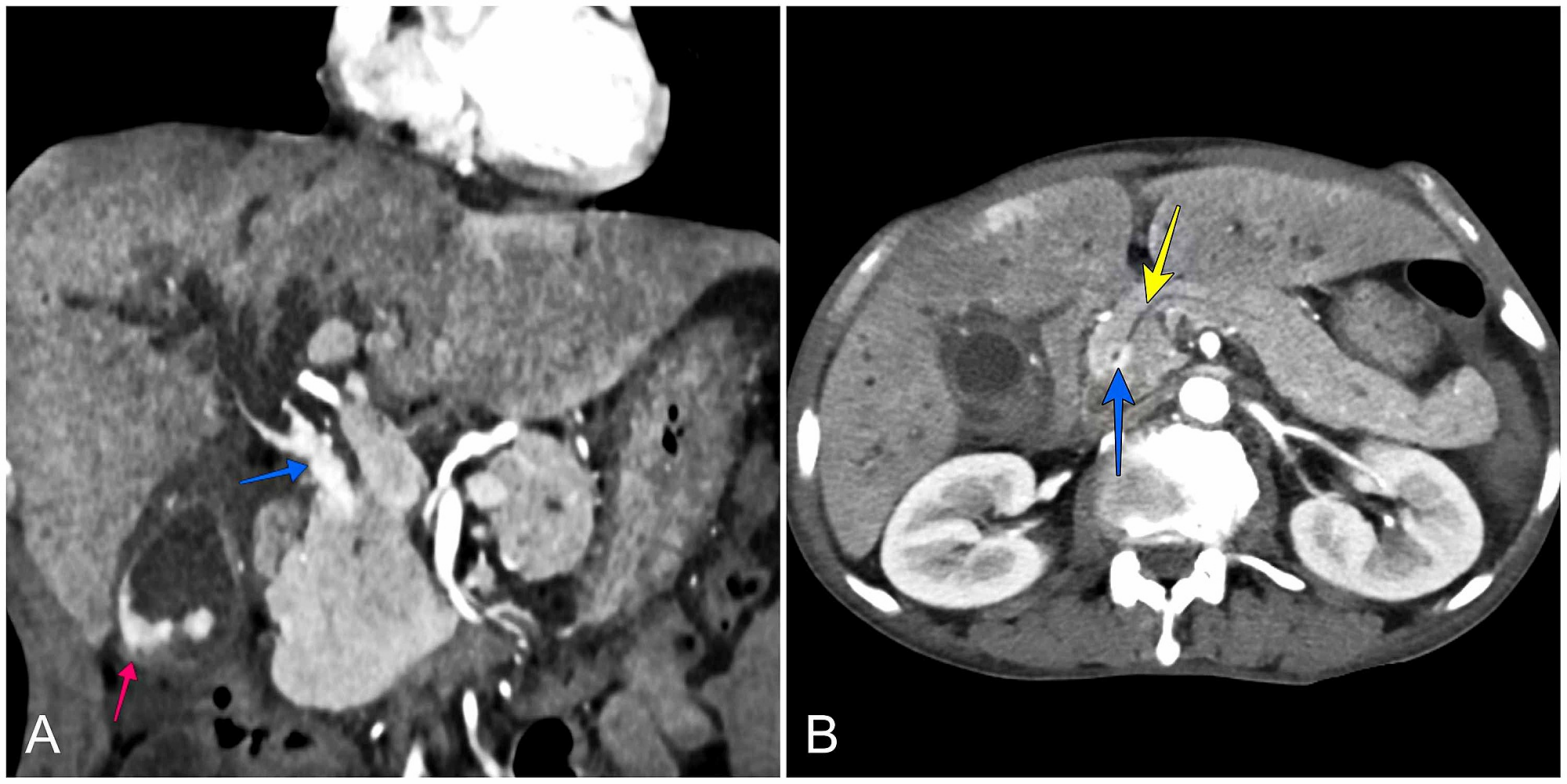
CECT images - arterial phase A: coronal section showing hyperenhancing growth in the gallbladder (red arrow) and the bile duct (blue arrow); B: axial section showing the pancreatic duct (yellow arrow) joining the common bile duct with a circumferential enhancing growth (blue arrow) in the head of the pancreas (outside the duodenal wall) CECT: contrast-enhanced computed tomography

A 60-year-old male patient presented with three months' history of progressive jaundice, high colored urine, and pale stools. Physical examination revealed right upper quadrant tenderness with no palpable organomegaly. Laboratory workup revealed cholestasis (direct bilirubin: 10.4 mg/dL; alkaline phosphatase: 1,093 U/L; and gamma-glutamyl transferase: 1,143 U/L). On contrast-enhanced CT (CECT) scan, there was an extensive hyperenhancing growth in the gallbladder and the bile duct (middle and distal) with upstream dilatation of the intrahepatic biliary radicles (Figure 1A). Ultrasound-guided fine-needle aspiration cytology was performed from the gallbladder mass, which revealed adenocarcinoma. One axial section of the scan also revealed the pancreatic duct to be joining the bile duct inside the head of the pancreas (Figure 1B).

**Figure 2:**
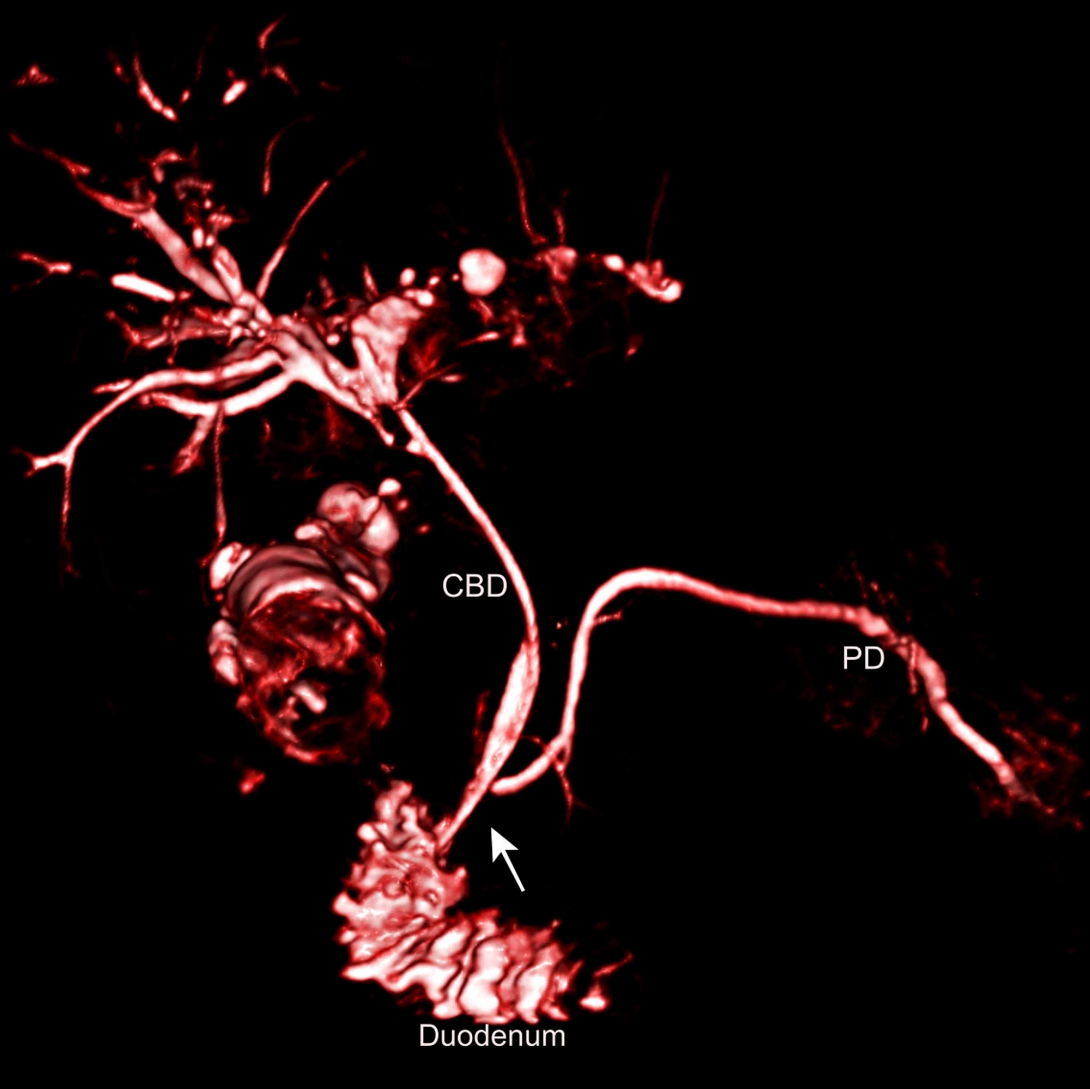
Volume reconstructed 3D MRCP image (done following bile duct stenting) The image showing the pancreatic duct (PD) joining at an acute angle with the common bile duct (CBD) outside the duodenal wall with a long common channel (white arrow) MRCP: magnetic resonance cholangiopancreatography

Following endoscopic biliary stenting, a magnetic resonance cholangiopancreatography (MRCP) scan was done, which confirmed the presence of an APBDJ with a long common channel of 16 mm (Figure 2).

There was a delay in performing elective operating services due to the coronavirus disease 2019 (COVID-19), and the patient, unfortunately, developed multiple liver metastases during his hospital stay. So no curative resection could be performed. Consequently, no pathological diagnosis could be attained for the APBDJ. After a discussion at the institutional multidisciplinary review board, he was started on a palliative chemotherapy regimen.

## Discussion

First described by Babbitt in 1969, APBDJ, also known as pancreaticobiliary maljunction, is a congenital malformation in which the pancreatic and bile ducts join outside the duodenal wall with an abnormally long common channel [6]. In 2015, the Japanese Study Group on Pancreaticobiliary Maljunction classified it into four types: (A) stenotic type, (B) non‐stenotic type, (C) dilated channel type, and (D) complex type [7]. This patient had a type B or non-stenotic type maljunction without any dilatation.

APBDJ has been reported to cause extensive double gallbladder-bile duct tumors [8]. A Japanese study has reported an incidence rate of 4.1% for such double bile duct cancers in patients with APBDJ without a biliary dilatation [9]. Biliary cancers develop 15-20 years earlier in patients with APBDJ [10]. Such a long common channel outside the duodenal wall results in the loss of the sphincter mechanism. Therefore, due to higher hydrostatic pressure in the pancreatic duct than the bile duct [11], there is a free flow of pancreatic secretions in the bile duct. The proposed mechanism of carcinogenesis is related to reflux and persistence of pancreatic juice in the biliary tract, resulting in increased intraductal pressure of the bile duct and activation of phospholipase A2 in the biliary tract. Phospholipase A2 converts bile lecithin to lysolecithin, which has a robust damaging effect on the entire biliary tract's cell membrane, including the gallbladder. Such repeated damage to epithelial cells accelerates the cell cycle, promoting DNA damage and carcinogenesis [12].

MRCP is the investigation of choice to demonstrate this anatomical anomaly of the pancreaticobiliary ducts, with a sensitivity of around 75% [13]. 3D volume reconstructed MRCP can provide a high-resolution real-life ductal representation of the aberrant anatomy along with revealing possible complications like a choledochal cyst, biliary stricture, or a suspicious occult malignancy. A secretin-stimulated dynamic MRCP has a higher sensitivity due to the ability to demonstrate finer ductal details by distending and enhancing even the smaller ducts and is useful in complicated APBDJ types [14]. A gadoxetic acid-enhanced MRI helps demonstrate biliopancreatic reflux in cases of doubtful diagnoses [15]. A multidetector CECT can also diagnose APBDJ with additional information of any associated malignancies, though its sensitivity is less than that of MRCP [16]. However, endoscopic retrograde cholangiopancreatography (ERCP) is the most effective method or the gold standard in diagnosing APBDJ. It can also demonstrate the lack of effect of the sphincter of Oddi at the ampulla, even when the common channel is not very long with a doubtful diagnosis. However, being an invasive investigation, it is not without complications. Common complications are pancreatitis, cholangitis, and bleeding. In APBDJ patients with biliary stricture, there is a higher risk of post-procedure cholangitis [17].

Though historically there were no evidence-based recommendations regarding the surgical treatment of patients incidentally detected with APBDJ, most consensus guidelines now recommend surgical treatment, irrespective of the presence or absence of symptoms, due to the definite risk of malignancy attributable to the ‘carcinogenic anatomical configuration.’ Prophylactic cholecystectomy, along with a biliary diversion procedure, is the surgical procedure of choice. If there is a congenital dilatation of the extrahepatic bile duct, it should be resected to remove the carcinogenic tissue [10].

## Conclusions

A patient with gallbladder cancer with obstructive jaundice generally implies an advanced disease stage and has a poor prognosis. However, a possibility of the synchronous double lesion should be kept in mind, which can have a much better prognosis than a locally advanced gallbladder cancer if approached with aggressive surgical treatment. ERCP is the gold standard for diagnoses; however, owing to its invasive nature, MRCP is the investigation of choice. APBDJ has definite carcinogenic potential and should be treated surgically, even when diagnosed incidentally.
